# Enhancing patient care: updated sedative choices in the intensive care unit

**DOI:** 10.62675/2965-2774.20240152-en

**Published:** 2024-11-14

**Authors:** Federico Carlos Carini, Mariana Luz, Dimitri Gusmao-Flores

**Affiliations:** 1 Hospital Italiano de Buenos Aires Intensive Care Unit Buenos Aires Argentina Intensive Care Unit, Hospital Italiano de Buenos Aires - Buenos Aires, Argentina.; 2 Hospital da Mulher Intensive Care Unit Salvador BA Brazil Intensive Care Unit, Hospital da Mulher - Salvador (BA), Brazil.; 3 Faculdade de Medicina da Bahia Salvador BA Brazil Postgraduate Program in Medicine and Health, Faculdade de Medicina da Bahia - Salvador (BA), Brazil.

## INTRODUCTION

Sedation plays a crucial role in the management of critically ill patients in the intensive care unit (ICU), aiming to alleviate discomfort, facilitate mechanical ventilation, and optimize patient care. However, achieving appropriate sedation levels while minimizing adverse effects remains a complex challenge. Over the years, there has been a paradigm shift toward more patient-centered approaches, emphasizing the importance of individualized sedation strategies and the avoidance of oversedation. This review provides an update on the current landscape of sedation management in the ICU, highlighting recent advancements and emerging trends.

### New scenario and old tools

It is interesting to consider that an update in the ICU usually refers to new strategies or tools. However, in the field of sedation in critical care, it can be seen as the application of old and well-known tools in a new scenario. The implementation of sedation protocols, the ABCDEF bundle, and targeted sedation management, among many others, are strategies that aim to reduce unnecessary sedative exposure and minimize the incidence of oversedation. Such strategies have been advocated in guidelines since the early 2000s. ^(1)^ Several studies have demonstrated the efficacy of sedation protocols in improving patient outcomes, including a shorter duration of mechanical ventilation and a shorter ICU length of stay.^(2)^ The most comprehensive example of such protocols or bundles is the ABCDEF bundle.^(3)^

The coronavirus disease 2019 (COVID-19) pandemic has likely led to the deadoption of some strategies with proven efficacy and the resurfacing of others, such as deep sedation.^(4)^ Sedative drugs must be reserved for specific situations in which sedation is part of the treatment (e.g., intracranial hypertension) or to control agitation when analgesia or nonpharmacological strategies fail because their inappropriate use can be harmful.

### Concerning sedative choice

A variety of pharmacological agents, each with unique pharmacokinetic and pharmacodynamic properties, are available for sedation in the ICU. One may wonder, is there any strong evidence that a class of sedatives is better than the others? Current clinical practice guidelines (CPGs) agree that the gold standard is to target conscious sedation (Richmond Agitation-Sedation Scale [RASS] score of −1/0) as much as possible. Additionally, CPGs prioritize first-line sedatives such as propofol and/or dexmedetomidine, and benzodiazepines have been relegated as a last resort because of concerns about the risks of accumulation, delayed clearance, *delirium*, and a longer stay in the ICU.^(5)^ However, they remain broadly used worldwide, with more than 80% of physicians reporting prescribing midazolam for sedation in the recent SAMDS study.^(4)^ As discussed in a seminal pro/con debate about benzodiazepines, the following question arises: Is it the drug or how we use it that worsens the prognosis?^(6)^

**Figure 1:**
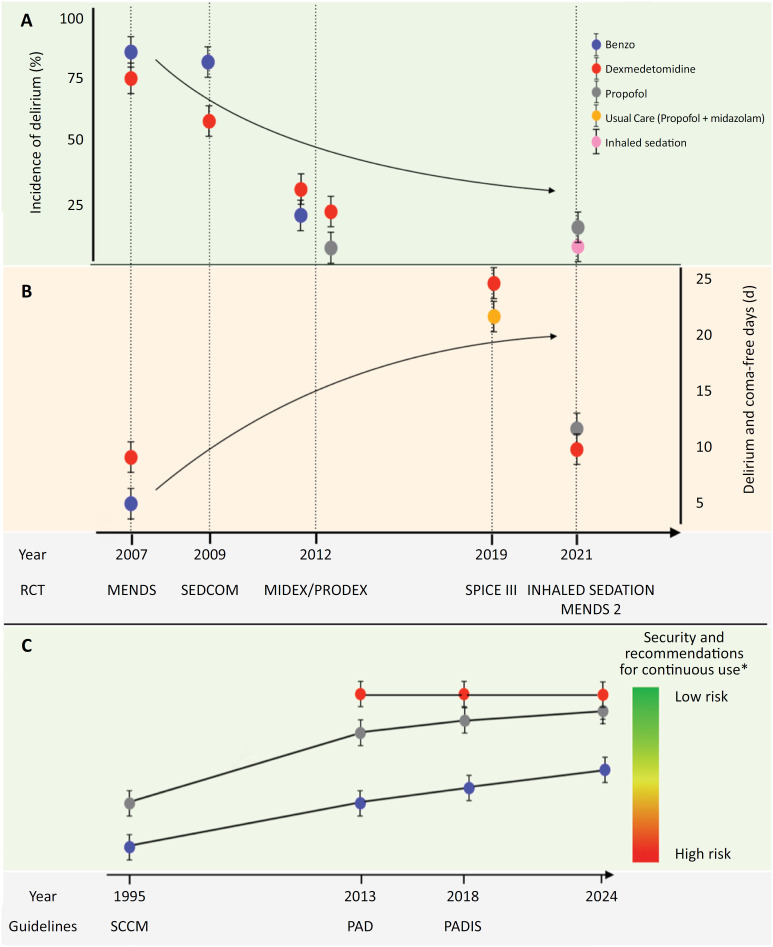
Timeline of evidence regarding the drug choice for sedation in the intensive care unit.

It is worth mentioning that this negative impact, initially observed with the use of lorazepam and then with midazolam, was consistently reduced, as analyzed sequentially in the MENDS trial (lorazepam *versus* dexmedetomidine), SEDCOM trial (midazolam *versus* dexmedetomidine), and MIDEX/PRODEX trial (midazolam or propofol *versus* dexmedetomidine).^(7-9)^ In each of these trials, this class of drug was progressively used in a more appropriate way, aligned with a sedation protocol that considered either low doses or interrupted the infusion. Therefore, rather than focusing only on a specific type of drug, it is perhaps more important to know how to use the drug effectively, especially in low-income centers where some sedatives may not be available. However, and according to current guidelines, benzodiazepines should be reserved for specific indications (status epilepticus, end-of-life care, etc.) or as a second- or third-line sedative in cases of difficult sedation.^(1)^ Figure 1 summarizes the timeline of the research presented here.

(A) During the evolution of the largest and most relevant trials on sedation, the incidence of *delirium* decreased. (B) Accordingly, *delirium* and coma-free days increased. (C) Recommendation regarding the continuous use of different sedatives. *The column on the right depicts the risk of the sedatives, including *delirium* and an extended time on mechanical ventilation: "green" indicates low risk and is therefore highly recommended; "red" indicates high risk and is not recommended. During the evolution of the guidelines, dexmedetomidine and propofol were the preferred classes of drugs (green zone: highly recommended, when necessary).

RCT - randomized clinical trial; MENDS - Maximizing the Efficacy of Targeted Sedation and Reducing Neurological Dysfunction; SEDCOM - Safety and Efficacy of Dexmedetomidine Compared with Midazolam; MIDEX - Midazolam with Dexmedetomidine; PRODEX - Propofol with Dexmedetomidine; SPICE - Sedation Practice in Intensive Care Evaluation; SCCM - Society of Critical Care Medicine; PAD - Pain, Agitation, and Delirium; PADIS - Pain, Agitation/Sedation, Delirium, Immobility and Sleep.

**References (Panel A/B):**

**1. MENDS:** Pandharipande PP, Pun BT, Herr DL, Maze M, Girard TD, Miller RR, et al. Effect of sedation with dexmedetomidine vs lorazepam on acute brain dysfunction in mechanically ventilated patients: the MENDS randomized controlled trial. JAMA. 2007;298(22):2644-53.

**2. SEDCOM:** Riker RR, Shehabi Y, Bokesch PM, Ceraso D, Wisemandle W, Koura F, Whitten P, Margolis BD, Byrne DW, Ely EW, Rocha MG; SEDCOM (Safety and Efficacy of Dexmedetomidine Compared With Midazolam) Study Group. Dexmedetomidine vs midazolam for sedation of critically ill patients: a randomized trial. JAMA. 2009;301(5):489-99.

**3. MIDEX/PRODEX:** Jakob SM, Ruokonen E, Grounds RM, Sarapohja T, Garratt C, Pocock SJ, Bratty JR, Takala J; Dexmedetomidine for Long-Term Sedation Investigators. Dexmedetomidine vs midazolam or propofol for sedation during prolonged mechanical ventilation: two randomized controlled trials. JAMA. 2012;307(11):1151-60.

**4. SPICE III:** Shehabi Y, Howe BD, Bellomo R, Arabi YM, Bailey M, Bass FE, Bin Kadiman S, McArthur CJ, Murray L, Reade MC, Seppelt IM, Takala J, Wise MP, Webb SA; ANZICS Clinical Trials Group and the SPICE III Investigators. Early sedation with dexmedetomidine in critically ill patients. N Engl J Med. 2019;380(26):2506-17.

**5. INHALED SEDATION:** Meiser A, Volk T, Wallenborn J, Guenther U, Becher T, Bracht H, Schwarzkopf K, Knafelj R, Faltlhauser A, Thal SC, Soukup J, Kellner P, Drüner M, Vogelsang H, Bellgardt M, Sackey P; Sedaconda study group. Inhaled isoflurane via the anaesthetic conserving device versus propofol for sedation of invasively ventilated patients in intensive care units in Germany and Slovenia: an open-label, phase 3, randomised controlled, non-inferiority trial. Lancet Respir Med. 2021;9(11):1231-40.

**6. MENDS 2:** Hughes CG, Mailloux PT, Devlin JW, Swan JT, Sanders RD, Anzueto A, Jackson JC, Hoskins AS, Pun BT, Orun OM, Raman R, Stollings JL, Kiehl AL, Duprey MS, Bui LN, O’Neal HR Jr, Snyder A, Gropper MA, Guntupalli KK, Stashenko GJ, Patel MB, Brummel NE, Girard TD, Dittus RS, Bernard GR, Ely EW, Pandharipande PP; MENDS2 Study Investigators. Dexmedetomidine or propofol for sedation in mechanically ventilated adults with sepsis. N Engl J Med. 2021;384(15):1424-36.

**References (Painel C):**

**7. SCCM 1995:** Shapiro BA, Warren J, Egol AB, Greenbaum DM, Jacobi J, Nasraway SA, et al. Practice parameters for intravenous analgesia and sedation for adult patients in the intensive care unit: an executive summary. Society of Critical Care Medicine. Crit Care Med. 1995;23(9):1596-600.

**8. PAD 2013:** Barr J, Fraser GL, Puntillo K, Ely EW, Gélinas C, Dasta JF, Davidson JE, Devlin JW, Kress JP, Joffe AM, Coursin DB, Herr DL, Tung A, Robinson BR, Fontaine DK, Ramsay MA, Riker RR, Sessler CN, Pun B, Skrobik Y, Jaeschke R; American College of Critical Care Medicine. Clinical practice guidelines for the management of pain, agitation, and delirium in adult patients in the intensive care unit. Crit Care Med. 2013;41(1):263-306.

**9. PADIS 2018:** Devlin JW, Skrobik Y, Gélinas C, Needham DM, Slooter AJ, Pandharipande PP, et al. Executive Summary: Clinical Practice Guidelines for the Prevention and Management of Pain, Agitation/Sedation, Delirium, Immobility, and Sleep Disruption in Adult Patients in the ICU. Crit Care Med. 2018;46(9):1532-48.

With respect to dexmedetomidine, the SPICE III trial showed heterogeneity of treatment effects by age when using dexmedetomidine versus standard sedation (lower 90-day mortality in patients older than 65 years and higher mortality in patients 65 years or younger). Further studies are needed to confirm these findings and to understand the underlying mechanisms involved.^(10)^

### Monitoring the sedation level

Accurate assessment of sedation depth is essential for tailoring sedation regimens to individual patient needs. When clinical evaluation is feasible, various sedation assessment tools are available, including the RASS and the Sedation-Agitation Scale (SAS). However, in patients requiring deep sedation (i.e., RASS score −4/-5) or neuromuscular blockade, clinical evaluation is not feasible. Newer technologies such as processed electroencephalography monitoring offer objective measures of sedation depth and may aid in optimizing sedation management. It has been recommended for all patients under deep sedation (regardless of neuromuscular blockade) when clinical evaluation is not feasible.^(11)^ However, the only recent systematic review evaluating its use in the ICU has not demonstrated benefits in sedated patients on mechanical ventilation.^(12)^

### Inhaled sedation

While inhaled sedation has historically been confined to the operating room, recent advancements have extended its applicability to the ICU setting. The main benefits are rapid clearance, no accumulation, a lower risk of *delirium*, and a shorter ICU stay.^(13)^ A recent noninferiority trial demonstrated that inhaled sedation is safe and efficacious in ICU patients.^(14)^ The implementation of this novel tool requires proper team training for setup and troubleshooting. However, there are areas of uncertainty, including long-term effects on patients and increased costs, which should be the focus of future research.

### Future directions

Despite advances in sedation management, several challenges remain. As mentioned, research into new drugs (e.g., remimazolam, inhaled sedation), devices for monitoring sedation and new protocols is essential to continue advancing this field. However, we feel that it is critical to double the efforts to enhance interdisciplinary collaboration and apply implementation science/knowledge translation strategies as essential steps in improving patient outcomes and optimizing resource utilization in the ICU.

In summary, the landscape of sedation management in the ICU continues to evolve, with ongoing efforts focused on optimizing existing strategies and integrating novel approaches. By embracing patient-centered care principles and leveraging advancements in monitoring technology, healthcare providers can navigate the complexities of sedation management to enhance patient outcomes in critical care settings.
