# Supplementary material for: Enhancing patient care: updated sedative choices in the intensive care unit
Source: Crit Care Sci. 2024 Nov 14;36:e20240152en. doi: 10.62675/2965-2774.20240152-en (PMC11634283; doi:10.62675/2965-2774.20240152-en)
Supplement: Translation

# Translation: Aprimorando a assistência ao paciente: opções atualizadas de sedativos na unidade de terapia intensiva

**DOI:** 10.62675/2965-2774.20240152-pt

**Authors:** Federico Carlos Carini, Mariana Luz, Dimitri Gusmao-Flores

**Affiliations:** 1 Buenos Aires Argentina Unidade de Terapia Intensiva, Hospital Italiano de Buenos Aires - Buenos Aires, Argentina.; 2 Salvador BA Brasil Unidade de Terapia Intensiva, Hospital da Mulher - Salvador (BA), Brasil.; 3 Salvador BA Brasil Programa de Pós-Graduação em Medicina e Saúde, Faculdade de Medicina da Bahia - Salvador (BA), Brasil.

## INTRODUÇÃO

A sedação desempenha papel fundamental no tratamento de pacientes críticos na unidade de terapia intensiva (UTI), com o objetivo de aliviar o desconforto, facilitar a ventilação mecânica e otimizar a assistência ao paciente. Entretanto, atingir níveis adequados de sedação e minimizar os efeitos adversos continua um desafio complexo. Ao longo dos anos, houve mudança de paradigma em direção a abordagens mais centradas no paciente, enfatizando a importância de estratégias de sedação individualizadas e a prevenção da sedação excessiva. Esta revisão fornece uma atualização sobre o panorama atual do manejo da sedação na UTI, destacando os avanços recentes e as tendências emergentes.

### Novo cenário, ferramentas antigas

É interessante considerar que uma atualização na UTI geralmente se refere a novas estratégias ou ferramentas. No entanto, no campo da sedação em cuidados intensivos, isso pode ser visto como a aplicação de ferramentas antigas e bem conhecidas em um novo cenário. A implementação de protocolos de sedação, o *bundle* ABCDEF e o manejo de sedação direcionado, entre muitos outros, são estratégias que visam reduzir a exposição desnecessária a sedativos e minimizar a incidência de sedação excessiva. Essas estratégias têm sido defendidas em diretrizes desde o início dos anos 2000.^(1)^ Vários estudos demonstraram a eficácia de protocolos de sedação na melhoria de desfechos de pacientes, incluindo menor duração da ventilação mecânica e menor tempo de internação na UTI.^(2)^ O exemplo mais abrangente de tais protocolos ou *bundles* é o ABCDEF.^(3)^

A pandemia da doença do coronavírus 2019 (COVID-19) provavelmente levou à desistência de algumas estratégias com eficácia comprovada e ao ressurgimento de outras, como a sedação profunda.^(4)^ Os medicamentos sedativos devem ser reservados para situações específicas em que a sedação faz parte do tratamento (por exemplo: hipertensão intracraniana) ou para controlar a agitação quando a analgesia ou as estratégias não farmacológicas falham, pois seu uso inadequado pode ser prejudicial.

### Com relação à escolha do sedativo

Uma variedade de agentes farmacológicos, cada um com propriedades farmacocinéticas e farmacodinâmicas exclusivas, está disponível para sedação na UTI. Podemos nos perguntar: há alguma evidência forte de que uma classe de sedativos seja melhor do que as outras? As diretrizes de prática clínica (DPCs) atuais concordam que o padrão-ouro é visar à sedação consciente (pontuação de −1/0 na escala de agitação-sedação de Richmond [RASS]) o máximo possível. Além disso, as DPCs priorizam os sedativos de primeira linha, como propofol e/ou a dexmedetomidina; os benzodiazepínicos foram relegados como último recurso devido a preocupações com os riscos de acúmulo, liberação tardia, *delirium* e internação mais longa na UTI.^(5)^ No entanto, eles continuam sendo amplamente utilizados em todo o mundo; mais de 80% dos médicos relatam a prescrição de midazolam para sedação no recente estudo SAMDS.^(4)^ Conforme discutido em um debate seminal pró/contra os benzodiazepínicos, surge a seguinte pergunta: É o medicamento ou a forma como o usamos que piora o prognóstico?^(6)^

Vale ressaltar que esse impacto negativo, inicialmente observado com o uso do lorazepam e, posteriormente, com o midazolam, foi consistentemente reduzido, conforme analisado sequencialmente nos estudos MENDS (lorazepam *versus* dexmedetomidina), no SEDCOM (midazolam *versus* dexmedetomidina) e no MIDEX/PRODEX (midazolam ou propofol *versus* dexmedetomidina).^(7-9)^ Em cada um desses estudos, essa classe de fármaco foi progressivamente usada de forma mais apropriada, alinhada a um protocolo de sedação que considerava doses baixas ou interrompia a infusão. Portanto, em vez de se concentrar apenas em um tipo específico de medicamento, talvez seja mais importante saber como usar o medicamento de forma eficaz, especialmente em centros de baixa renda, onde alguns sedativos podem não estar disponíveis. No entanto, e de acordo com as diretrizes atuais, os benzodiazepínicos devem ser reservados para indicações específicas (*status epilepticus*, cuidados de fim de vida, etc.) ou como sedativo de segunda ou terceira linha em casos de sedação difícil.^(1)^ A figura 1 resume a linha do tempo da pesquisa apresentada aqui.

**Figura 1:** Linha do tempo das evidências da escolha do medicamento para sedação na unidade de terapia intensiva.

(A) Durante a evolução dos maiores e mais relevantes estudos sobre sedação, a incidência de *delirium* diminuiu. (B) Consequentemente, os dias sem *delirium* e coma aumentaram. (C) Recomendação do uso contínuo de diferentes sedativos. *A coluna da direita mostra o risco dos sedativos, incluindo *delirium* e tempo prolongado de ventilação mecânica: verde indica baixo risco e, portanto, é altamente recomendado; vermelho indica alto risco e não é recomendado. Durante a evolução das diretrizes, a dexmedetomidina e o propofol foram as classes preferidas de medicamentos (zona verde: altamente recomendado, quando necessário).

ECR - ensaio clínico randomizado; MENDS - *Maximizing the Efficacy of Targeted Sedation and Reducing Neurological Dysfunction*; SEDCOM - *Safety and Efficacy of Dexmedetomidine Compared with Midazolam*; MIDEX - *Midazolam with Dexmedetomidine*; PRODEX - *Propofol with Dexmedetomidine*; SPICE - *Sedation Practice in Intensive Care Evaluation*; SCCM - *Society of Critical Care Medicine*; PAD - *Pain, Agitation, and Delirium*; PADIS - *Pain, Agitation/Sedation, Delirium, Immobility and Sleep*.

**Referências (Painel A/B):**

**1. MENDS:** Pandharipande PP, Pun BT, Herr DL, Maze M, Girard TD, Miller RR, et al. Effect of sedation with dexmedetomidine vs lorazepam on acute brain dysfunction in mechanically ventilated patients: the MENDS randomized controlled trial. JAMA. 2007;298(22):2644-53.

**2. SEDCOM:** Riker RR, Shehabi Y, Bokesch PM, Ceraso D, Wisemandle W, Koura F, Whitten P, Margolis BD, Byrne DW, Ely EW, Rocha MG; SEDCOM (Safety and Efficacy of Dexmedetomidine Compared With Midazolam) Study Group. Dexmedetomidine vs midazolam for sedation of critically ill patients: a randomized trial. JAMA. 2009;301(5):489-99.

**3. MIDEX/PRODEX:** Jakob SM, Ruokonen E, Grounds RM, Sarapohja T, Garratt C, Pocock SJ, Bratty JR, Takala J; Dexmedetomidine for Long-Term Sedation Investigators. Dexmedetomidine vs midazolam or propofol for sedation during prolonged mechanical ventilation: two randomized controlled trials. JAMA. 2012;307(11):1151-60.

**4. SPICE III:** Shehabi Y, Howe BD, Bellomo R, Arabi YM, Bailey M, Bass FE, Bin Kadiman S, McArthur CJ, Murray L, Reade MC, Seppelt IM, Takala J, Wise MP, Webb SA; ANZICS Clinical Trials Group and the SPICE III Investigators. Early sedation with dexmedetomidine in critically ill patients. N Engl J Med. 2019;380(26):2506-17.

**5. Inhaled SedatioN:** Meiser A, Volk T, Wallenborn J, Guenther U, Becher T, Bracht H, Schwarzkopf K, Knafelj R, Faltlhauser A, Thal SC, Soukup J, Kellner P, Drüner M, Vogelsang H, Bellgardt M, Sackey P; Sedaconda study group. Inhaled isoflurane via the anaesthetic conserving device versus propofol for sedation of invasively ventilated patients in intensive care units in Germany and Slovenia: an open-label, phase 3, randomised controlled, non-inferiority trial. Lancet Respir Med. 2021;9(11):1231-40.

**6. MENDS 2:** Hughes CG, Mailloux PT, Devlin JW, Swan JT, Sanders RD, Anzueto A, Jackson JC, Hoskins AS, Pun BT, Orun OM, Raman R, Stollings JL, Kiehl AL, Duprey MS, Bui LN, O’Neal HR Jr, Snyder A, Gropper MA, Guntupalli KK, Stashenko GJ, Patel MB, Brummel NE, Girard TD, Dittus RS, Bernard GR, Ely EW, Pandharipande PP; MENDS2 Study Investigators. Dexmedetomidine or propofol for sedation in mechanically ventilated adults with sepsis. N Engl J Med. 2021;384(15):1424-36.

**Referências (Painel C):**

**1. SCCM 1995:** Shapiro BA, Warren J, Egol AB, Greenbaum DM, Jacobi J, Nasraway SA, et al. Practice parameters for intravenous analgesia and sedation for adult patients in the intensive care unit: an executive summary. Society of Critical Care Medicine. Crit Care Med. 1995;23(9):1596-600.

**2. PAD 2013:** Barr J, Fraser GL, Puntillo K, Ely EW, Gélinas C, Dasta JF, Davidson JE, Devlin JW, Kress JP, Joffe AM, Coursin DB, Herr DL, Tung A, Robinson BR, Fontaine DK, Ramsay MA, Riker RR, Sessler CN, Pun B, Skrobik Y, Jaeschke R; American College of Critical Care Medicine. Clinical practice guidelines for the management of pain, agitation, and delirium in adult patients in the intensive care unit. Crit Care Med. 2013;41(1):263-306.

**3. PADIS 2018:** Devlin JW, Skrobik Y, Gélinas C, Needham DM, Slooter AJ, Pandharipande PP, et al. Executive Summary: Clinical Practice Guidelines for the Prevention and Management of Pain, Agitation/Sedation, Delirium, Immobility, and Sleep Disruption in Adult Patients in the ICU. Crit Care Med. 2018;46(9):1532-48.

Com relação à dexmedetomidina, o estudo SPICE III mostrou heterogeneidade dos efeitos do tratamento por idade ao usar a dexmedetomidina em comparação com a sedação padrão (menor mortalidade em 90 dias em pacientes com mais de 65 anos e maior mortalidade em pacientes com 65 anos ou menos). São necessários mais estudos para confirmar esses achados e entender os mecanismos subjacentes envolvidos.^(10)^

### Monitoramento do nível de sedação

A avaliação precisa da profundidade da sedação é essencial para adaptar os esquemas de sedação às necessidades individuais do paciente. Quando a avaliação clínica é viável, várias ferramentas de avaliação da sedação estão disponíveis, incluindo a RASS e a Escala de Sedação-Agitação (SAS). Entretanto, em pacientes que necessitam de sedação profunda (ou seja, pontuação RASS −4/-5) ou bloqueio neuromuscular, a avaliação clínica não é viável. Tecnologias mais recentes, como o monitoramento de eletroencefalografia processada, oferecem medidas objetivas da profundidade da sedação e podem ajudar a otimizar o manejo da sedação. Ela tem sido recomendada para todos os pacientes sob sedação profunda (independentemente do bloqueio neuromuscular) quando a avaliação clínica não é viável.^(11)^ Entretanto, a única revisão sistemática recente que avaliou seu uso na UTI não demonstrou benefícios em pacientes sedados em ventilação mecânica.^(12)^

### Sedação inalatória

Embora a sedação inalatória tenha sido historicamente confinada à sala de cirurgia, os avanços recentes estenderam sua aplicabilidade ao ambiente da UTI. Os principais benefícios são a liberação rápida, a ausência de acúmulo, o menor risco de *delirium* e a internação mais curta na UTI.^(13)^ Um estudo recente de não inferioridade demonstrou que a sedação inalatória é segura e eficaz em pacientes de UTI.^(14)^ A implantação dessa nova ferramenta requer treinamento adequado da equipe para a configuração e solução de problemas. No entanto, há áreas de incerteza, incluindo efeitos de longo prazo nos pacientes e aumento de custos, que devem ser a prioridade de pesquisas futuras.

### Orientações futuras

Apesar dos avanços no manejo da sedação, vários desafios permanecem. A pesquisa de novos medicamentos (por exemplo, remimazolam, sedação inalatória), dispositivos para monitorar a sedação e novos protocolos são essenciais para continuar avançando nesse campo. No entanto, acreditamos que é fundamental dobrar os esforços para aprimorar a colaboração interdisciplinar e aplicar estratégias de implementação científica/tradução de conhecimento como etapas essenciais para melhorar os desfechos de pacientes e otimizar a utilização de recursos na UTI.

Em resumo, o cenário do manejo da sedação na UTI continua a evoluir, com esforços contínuos focados na otimização das estratégias existentes e na integração de novas abordagens. Ao adotar princípios de cuidados centrados no paciente e aproveitar os avanços na tecnologia de monitoramento, os profissionais de saúde podem navegar pelas complexidades do manejo da sedação para melhorar os desfechos de pacientes em ambientes de cuidados intensivos.
